# The telomeric PARP, tankyrases, as targets for cancer therapy

**DOI:** 10.1038/sj.bjc.6602951

**Published:** 2006-01-17

**Authors:** H Seimiya

**Affiliations:** 1 Division of Molecular Biotherapy, Cancer Chemotherapy Center, Japanese Foundation for Cancer Research, 3-10-6 Ariake, Koto-ku, Tokyo 135-8550, Japan

**Keywords:** tankyrase, poly(ADP-ribosyl)ation, telomeres, telomerase

## Abstract

The requirement for the maintenance of telomeres by telomerase by most cancer cells for continued proliferation is a target in anticancer strategies. Tankyrases are poly(ADP-ribose) polymerases that enhance telomerase access to telomeres. Tankyrase 1 modulates telomerase inhibition in human cancer cells and is reviewed in this report as a potential telomere-directed anticancer target.

Telomeres, the protective DNA–protein complexes at eukaryotic chromosome ends, are distinguished from DNA double-strand breaks, which would otherwise cause nonhomologous end joining or homologous recombination. DNA replication machinery cannot replicate the very ends of chromosomal DNA, known as the end replication problem, and this would result in the gradual loss of telomeres after each round of DNA replication, unless new telomeric DNA was synthesized by telomerase. The level of telomerase activity in most human somatic cells is so weak that telomere attrition is unavoidable. Critically shortened telomeres are recognized as damaged DNA (d'Adda di Fagagna *et al*, 2003), and this leads to replicative cell senescence. As a result, telomeres are often referred to as a ‘mitotic clock’ that predetermines the replicative capacity of mortal cells. In contrast with human somatic cells, up to 90% of all human cancer cells have a high level of telomerase activity that maintains telomere length (Kim *et al*, 1994).

Telomerase consists of a ubiquitously expressed RNA template, TR (or TERC), and a catalytic subunit, TERT, the expression of which is the limiting factor for the enzyme's activity. TERT expression correlates positively with cellular immortalization and carcinogenesis. Introduction of dominant-negative TERT mutants into cancer cells shortens their telomeres and induces subsequent apoptosis (Hahn *et al*, 1999; Zhang *et al*, 1999). The activation of telomerase by most cancer cells effectively bypasses replicative senescence. Accordingly, telomerase has been promoted as a potential target in anticancer strategies, and various telomerase inhibitors have been reported (Seimiya *et al*, 2002 and references therein). This minireview describes some potential problems with telomerase inhibitors and introduces the telomeric poly(ADP-ribose) polymerases (PARP), tankyrase 1 and 2 (Figure 1), as the second potential target for telomere-directed molecular cancer therapeutics.

## TELOMERE ELONGATION BY TANKYRASE 1

In telomerase-positive cells, average telomere length is stabilized by a ‘protein-counting’ mechanism, in which a series of telomere-associated proteins negatively regulate telomere elongation by telomerase (reviewed in Smogorzewska and de Lange, 2004). Longer telomeres have greater numbers of TRF1, a double-stranded telomeric repeat binding protein. In conjunction with the downstream TIN2-TPP1-POT1 telomere-associated complex, TRF1 blocks access of telomerase to telomeres (Figure 2).

**Figure 1:**
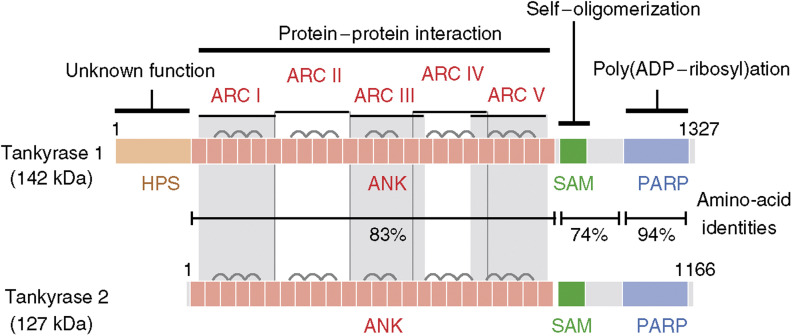
Structures of tankyrase 1 and tankyrase 2. HPS, homopolymeric runs of His, Pro, and Ser, without known functions; ANK, ankyrin domain, consisting of 24 ANK repeats; SAM, multimerization domain homologous to the sterile alpha motif; PARP, PARP catalytic domain that adds ADP-ribose chains onto acceptor proteins. The ANK domain is further divided into five well-conserved ANK repeat clusters (ARC), each of which contributes to ligand binding. Bridges above two adjacent ANK repeats indicate the presence of a conserved histidine contributing to inter-repeat stabilization.

Tankyrase 1 (TRF1-interacting ankyrin-related ADP-ribose polymerase 1) was originally identified as a TRF1-binding protein by using a yeast two-hybrid screen (Smith *et al*, 1998). This 140-kDa protein consists of four characteristic domains (Figure 1): the N-terminus is known as the HPS domain, containing homopolymeric runs of histidine, proline, and serine. The functional significance of the HPS domain is unknown. Tankyrase 1 is also comprised of an ANK domain placing the protein within the ANK family of proteins. As in the original ankyrins, the tankyrase 1 ANK domain is composed of a long stretch of 24 ANK repeats, providing a platform for protein–protein interactions. Unlike the ANK of ankyrins, the ANK domain of tankyrase 1 is further divided into five well-conserved subdomains (Seimiya and Smith, 2002; De Rycker *et al*, 2003). Each subdomain, designated as ARC (ANK repeat cluster) I–V, works as an independent TRF1-binding site. TRF1 recognition by the most C-terminal subdomain ARC V is the most important for the telomeric function of tankyrase 1 (Seimiya and Smith, 2002; Seimiya *et al*, 2004). The sterile alpha motif (SAM) domain, adjacent to the ARC V domain, contributes to multimerization of tankyrase 1 (De Rycker *et al*, 2003; De Rycker and Price, 2004). The most striking feature of tankyrase 1 is the C-terminal PARP domain, which catalyses poly(ADP-ribosyl)ation of acceptor proteins using NAD as a substrate. This post-translational modification provides significant negative charges to the acceptor proteins and often disrupts interactions between the acceptor proteins and the DNA. In general, poly(ADP-ribosyl)ation is involved in various physiological events, such as DNA replication, DNA repair, gene expression, chromatin decondensation, malignant transformation, cellular differentiation, and apoptosis.

Tankyrase 1 recognizes TRF1 via the ARC subdomains within the ANK domain and is localized at the telomeres (Smith *et al*, 1998; Cook *et al*, 2002; Seimiya and Smith, 2002). TRF1 is poly(ADP-ribosyl)ated by tankyrase 1, and this modification blocks the ability of TRF1 to bind telomeric DNA *in vitro* (Smith *et al*, 1998). In intact human cells, tankyrase 1 releases TRF1 from telomeres in a PARP activity-dependent manner (Smith and de Lange, 1999; Cook *et al*, 2002). Released TRF1 is rapidly ubiquitinated and subjected to proteasomal degradation (Chang *et al*, 2003), thereby explaining the striking linkage between poly(ADP-ribosyl)ation and protein degradation. Consistent with these observations, telomere elongation is induced by the overexpression of exogenous tankyrase 1 in the nucleus of telomerase-positive cells (Smith and de Lange, 2000). Also confirming the above findings, tankyrase 1-mediated telomere elongation is not observed in the absence of telomerase activity (Cook *et al*, 2002; Chang *et al*, 2003; Seimiya *et al*, 2005). Thus, by enhancing telomere access to telomerase, tankyrase 1 works as a positive regulator for telomere elongation by telomerase. This leads to the supposition that the action of telomerase at the telomere may be regulated by inhibitors of telomerase and also by molecules that regulate tankyrase 1, and hence telomere structure and telomerase access to the telomere (see below).

Tankyrase 2 is a closely related homologue of tankyrase 1 (see below). Tankyrase 1 forms a ternary complex with TRF1 and another TRF1-binding protein, TIN2. In this complex, poly(ADP-ribosyl)ation of TRF1 is prevented by TIN2 (Ye and de Lange, 2004). The mechanisms underlying tankyrase activation or inhibition are unknown. It is known that tankyrase 1 is not activated by damaged DNA, which is an activator of PARP-1, the most abundant member among the PARP family (Cook *et al*, 2002).

## TANKYRASES AS TARGETS FOR CANCER THERAPY

As described above, telomere maintenance by telomerase is the Achilles' heel of infinite growth for most cancer cells (Hahn *et al*, 1999; Zhang *et al*, 1999). Continuous treatment of cancer cells with telomerase inhibitors shortens telomeres and eventually induces cellular senescence and/or apoptosis (Seimiya *et al*, 2002 and references therein). Thus, according to this simple scenario, telomerase inhibitors have the potential to benefit cancer patients in the future.

A potential disadvantage is that telomere shortening depends on the repetitive occurrence of the DNA end replication problem resulting from cell division. For this reason, it is essential that telomerase inhibitors are not cytotoxic. Furthermore, as telomere loss is a gradual process there is a lag between the time telomerase is inhibited and the time telomeres shorten sufficiently to disrupt the capping function. This would necessitate long treatment schedules that may lead to acquired drug resistance both in the cell and throughout the body. In general, longer telomeres provide more binding sites for TRF1, which blocks telomere access to telomerase. Accordingly, telomere shortening *per se* compromises the effect of telomerase inhibitors since shorter telomeres have fewer TRF1 molecules and therefore allow easier access to residual telomerase activity (Seimiya *et al*, 2005). Thus, the rate of telomere shortening per cell division decreases with telomere shortening itself. This phenomenon results from the incomplete shutdown of telomerase activity by telomerase inhibitors. A better therapeutic outcome may result from increasing the efficiency of telomere shortening to hasten the telomere crisis.

**Figure 2:**
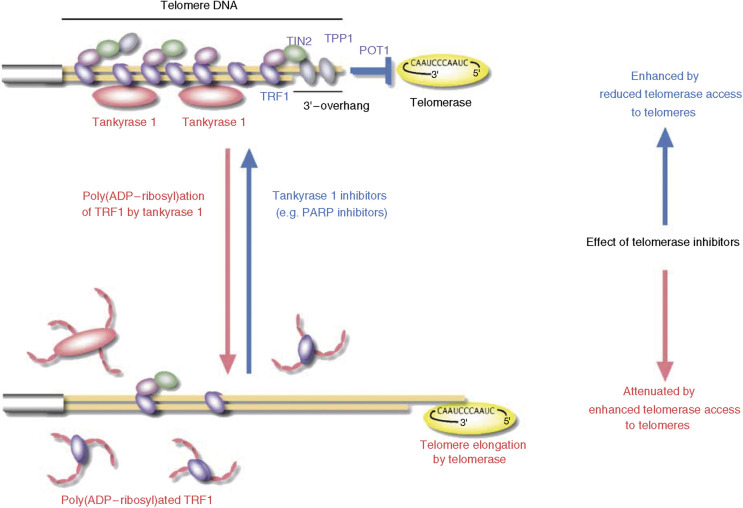
Telomere elongation by tankyrase 1 and impact on telomerase inhibitors. For telomere elongation, active telomerase needs to gain access to the telomeric 3′-overhang. The TRF1-TIN2-TPP1-POT1 telomeric protein complex limits telomerase access, whereas tankyrase 1 removes the telomeric protein complex by poly(ADP-ribosyl)ating TRF1. Either telomere shortening or tankyrase 1 upregulation, each of which decreases the TRF1-TIN2-TPP1-POT1 loading on a chromosome end, attenuates the impact of telomerase inhibitors by allowing access of residual telomerase activity. Conversely, blockade of tankyrase 1 enhances the effect of telomerase inhibitors. The relative importance of tankyrase 1 *vs* tankyrase 2 inhibition remains unclear.

Telomere accessibility is also a potential target for telomerase inhibition. Inhibition of tankyrases, that enhance telomerase access to telomeres, may indirectly induce cancer cell senescence by abrogating telomerase activity. Support for this rational is provided by the finding that tankyrase 1 confers resistance to telomerase inhibitors (Figure 2). In these experiments cells overexpressing tankyrase 1, which removes TRF1 from telomere DNA, have unchanged telomere length following treatment with the telomerase inhibitor, MST-312. This drug resistance is reversed by several known PARP inhibitors, such as 3-aminobenzamide (3AB) and PJ-34, which are able to block tankyrase 1 PARP activity (Seimiya *et al*, 2005). Even in cells that do not overexpress exogenous tankyrase 1 (but do express endogenous tankyrase 1), these PARP inhibitors enhance the rate of telomere shortening by means of a telomerase inhibitor, MST-312 (Seimiya *et al*, 2002), and induce earlier cell crisis. These PARP inhibitors do not directly inhibit telomerase activity but lead to telomere shortening, to a small extent, presumably by reducing telomere access to telomerase. Furthermore, MST-312 resistance caused by telomere shortening *per se* is reversed by 3AB. By comparison, 3AB has no effect on telomere length in telomerase-independent ALT (alternative lengthening of telomeres)-type cells, which maintain their telomere length by DNA recombination. Also, telomere shortening caused by an inherent end replication problem in normal fibroblasts is not accelerated by 3AB (Seimiya *et al*, 2005). Thus, it is expected that the effect of such PARP inhibitors on telomere length is selective to telomerase-positive cells. These observations provide support for tankyrase 1 as a suitable target for telomere-directed cancer therapy. Pathologically, tankyrase 1 gene expression is elevated in some tumours but not in others (Gelmini *et al*, 2004 and references in therein).

Another avenue for telomerase inhibition has recently emerged. Li *et al* (2004) reported that knockdown of the hTR telomerase RNA component by RNA interference (RNAi) induces a rapid antiproliferative effect on telomerase-positive cancer cells. Unexpectedly, this effect occurs without telomere attrition and is thereby independent of the initial telomere length of the target cells. These observations suggest that telomerase inhibition has bimodal effects on human cancer cells and that telomerase inhibitors may exert a more acute therapeutic effect than expected.

## OTHER FACES OF TANKYRASES

Multiple functions of tankyrases in accordance with a variety of binding partners pose the next challenging question about potential side effects of tankyrase-directed cancer therapy. Tankyrase 1 is also present at nontelomeric loci, including mitotic centrosomes, nuclear pore complexes, and Golgi apparatus (Smith and de Lange, 1999; Chi and Lodish, 2000). Furthermore, tankyrase 1 has a closely related homologue, tankyrase 2 that unlike tankyrase 1 lacks HPS domain. Tankyrase 1 is relatively abundant in reproductive tissues (i.e. testis and ovary), whereas the expression of tankyrase 2 is ubiquitous (Smith *et al*, 1998; Kaminker *et al*, 2001; Lyons *et al*, 2001; Cook *et al*, 2002). The functional difference and redundancy between the two proteins remain unknown.

Nontelomeric tankyrase 1/2-binding partners include insulin-responsive aminopeptidase (IRAP) (Chi and Lodish, 2000), the Grb14 signalling adaptor protein (Lyons *et al*, 2001), the 182 kDa tankyrase-binding protein (TAB182) (Seimiya and Smith, 2002), the nuclear/mitotic apparatus protein (NuMA) (Sbodio and Chi, 2002; Chang *et al*, 2005b), the Mcl-1 apoptotic regulator (Bae *et al*, 2003), and the Epstein–Barr virus nuclear antigen-1 (EBNA-1) (Deng *et al*, 2005). So far, TRF1, IRAP, TAB182, NuMA, EBNA-1 and tankyrase 1 and 2 are poly(ADP-ribosyl)ated by tankyrases.

The Golgi tankyrase 1 colocalizes with the glucose transporter GLUT4 vesicles where tankyrase 1 is associated with IRAP (Chi and Lodish, 2000). In insulin-stimulated adipocytes, tankyrase 1 is phosphorylated at serine residues by the mitogen-activated protein kinase pathway. Phosphorylation of tankyrase 1 results in upregulation of its intrinsic PARP activity (Chi and Lodish, 2000). Although the function of tankyrase 1 at the Golgi is unclear, the artificial formation of tankyrase 1-containing vesicles disrupts Golgi structure and inhibits apical secretion (De Rycker and Price, 2004).

During mitosis, tankyrase 1 is concentrated around the pericentriolar matrices (Smith and de Lange, 1999) in a NuMA-dependent manner (Chang *et al*, 2005b). NuMA plays an essential role in organizing microtubules at the spindle poles. As NuMA is poly(ADP-ribosyl)ated by tankyrase 1 during mitosis (Chang *et al*, 2005b), it is possible that tankyrase 1 regulates NuMA's function at the spindle poles. Interestingly, poly(ADP-ribosyl)ation is required for spindle assembly and structure (Chang *et al*, 2004), and tankyrase 1 is a key player in these processes (Chang *et al*, 2005a). Another fraction of tankyrase 1 remains at telomeres during mitosis (Smith *et al*, 1998) and is thought to play a role in sister chromatid resolution at telomeres. Support for this role of tankyrase 1 was provided by the metaphase arrest of cell division in tankyrase 1 knockdown experiments in which pairs of sister chromatids remain associated only at telomeres (Dynek and Smith, 2004). Recently, metaphase arrest by tankyrase 1 knockdown has been reported by another group, who shows intact sister chromatid cohesion, instead of telomeric cohesion, in tankyrase 1 knockdown cells (Chang *et al*, 2005a).

The protein structure of tankyrases suggests they act as scaffolding molecules. First, each of the five ARC subdomains works as an independent recognition site for tankyrase-binding proteins. This suggests that even a single tankyrase molecule can interact with multiple binding partners (Seimiya and Smith, 2002; Seimiya *et al*, 2004). Secondly, the SAM domain multimerizes tankyrases in an auto-poly(ADP-ribosyl)ation-sensitive manner. This multimerization presumably leads to assembly of a larger molecular lattice (De Rycker *et al*, 2003; De Rycker and Price, 2004) and may explain why tankyrase-binding proteins often localize to higher order intracellular structures, such as telomeres (TRF1), Golgi (IRAP), spindle poles (NuMA), and cortical actin (TAB182).

It is intriguing that murine TRF1 lacks the tankyrase recognition consensus site, RXX(P/A)DG, suggesting that the telomeric function of tankyrases is not conserved in mice (Sbodio and Chi, 2002). Other reported functions of tankyrases include involvement in apoptosis (Bae *et al*, 2003) and episomal regulation of Epstein–Barr virus OriP (origin of plasmid) (Deng *et al*, 2005). Taken together, these observations suggest an expanding network of tankyrase-mediated biological processes.

## CONCLUDING REMARKS

The pharmacological targeting of tankyrase 1 is a potentially significant anticancer strategy if used in conjunction with inhibitors of telomerase. This trend would further promote development not only of telomerase inhibitors but also of PARP inhibitors. In fact, recently, PARP inhibitors have been shown to be powerful against DNA repair-deficient tumours with the advantage of low cytotoxicity (Bryant *et al*, 2005; Farmer *et al*, 2005). PARP inhibitors are also implicated in other PARP-related diseases, such as stroke, myocardial ischaemia, diabetes, and central nervous system injury. Tankyrases also have multiple functions and we have to consider the potential side effects resulting from their inhibition. It is possible that the function of tankyrase 1 in mitosis (Dynek and Smith, 2004; Chang *et al*, 2005a) is less affected by PARP inhibitors than its function in maintenance of telomere length as mitosis is unaffected in PARP inhibitor-treated cells (Seimiya *et al*, 2005). At the physiological level, mechanisms for tankyrase regulation and the functional redundancy between tankyrase 1 and 2 are largely unknown. Future experiments in tankyrase 1 and 2 knockout animals may elucidate the mechanisms underlying the many functions of the tankyrase enzymes.
